# Clinical profile of dengue patients presenting with epistaxis at a tertiary hospital in Kathmandu, Nepal

**DOI:** 10.1186/s12879-026-14134-4

**Published:** 2026-07-31

**Authors:** Sabin Thapaliya, Bishal Budha, Sunil Gyawali, Dijesh Maskey, Saket Jha

**Affiliations:** 1 Department of Internal Medicine, Tribhuvan University Teaching Hospital, Maharajgunj, Kathmandu Nepal; 2 Maharajgunj Medical Campus, Institute of Medicine, Tribhuvan University Teaching Hospital, Maharajgunj, Kathmandu 44600 Nepal

**Keywords:** Dengue fever, Epistaxis, Warning signs, Nepal

## Abstract

**Background:**

Dengue fever poses a major global public health challenge, with a rising burden in countries like Nepal, where it can progress to severe dengue. Severe dengue, as defined by WHO criteria, manifests with critical plasma leakage leading to shock, severe hemorrhagic complications, and organ impairment. Epistaxis, though not a classic presenting symptom, may reflect underlying coagulopathy and signal heightened disease severity. However, the demographic profile, clinical characteristics, and outcomes of dengue patients presenting with epistaxis remain undescribed in Nepal. This study describes the clinical and laboratory profile of such patients admitted to a tertiary care hospital.

**Methods:**

We performed a retrospective observational study based on hospital records of patients admitted to Tribhuvan University Teaching Hospital, Kathmandu, Nepal, between 1st September 2022 and 30th November 2024. Patients with NS1-positive dengue fever who developed epistaxis during the course of illness were included. Data on demographics, comorbidities, prior medication use, timing of epistaxis, hospital stay, additional bleeding manifestations, hematological parameters (platelet count, WBC count, PT/INR), liver enzymes (AST/ALT), and transfusion requirements were analyzed.

**Result:**

A total of 27 patients with dengue fever and epistaxis were evaluated. The mean age was 42 years, and the majority (70.37%) were male. Epistaxis was the initial presentation among 59.26% of patients, while 40.74% developed it during hospitalization. Epistaxis typically occurred around day 4–7 of illness. At the time of epistaxis, the mean WBC count was 5462.96/µL (range 1,600 − 14,700/µL), and the mean platelet count was around 30,000/µL. /µL. Decreasing trend of WBC counts and an increasing trend of platelet counts were seen in the majority of patients. AST and ALT were elevated (mean AST 424 U/L, ALT: 166 U/L). One-third of patients experienced additional bleeding, most commonly from oral mucosa, followed by GI bleeding. Overall, 62.96% required transfusion, and the mean platelet transfusion was about 4 pints. Among those with bleeding from other sites, 89% required transfusion, highlighting the increased severity in this subgroup.

**Conclusion:**

Epistaxis in dengue patients most commonly occurs during the critical phase of illness and is associated with severe thrombocytopenia, hepatic dysfunction, and an increased risk of multisite bleeding. Although not all patients require transfusion, epistaxis may serve as an early clinical marker of severe dengue, underscoring the need for vigilant monitoring and timely intervention to prevent complications.

**Clinical trail:**

Not applicable.

## Background

Dengue virus (DENV) belongs to a group of enveloped viruses, Flaviruses which also includes West Nile virus and yellow fever virus, and involves arthropods as their vectors [1]. Dengue disease can be caused by 1 of 4 distinct but closely related dengue viruses and specifically are transmitted by mosquito of the genus Aedes (*Aedes aegypti*,* Aedes albopticus*) [2, 3]. The disease is now consistently present over 100 countries in the tropical and subtropical regions of Africa, South East Asia, the Eastern Mediterranean and Western Pacific and has more than 100 million symptomatic cases each year [2, 3]. 

Nepal reported first Dengue case in 2004 in a traveler [4]. Originally limited to lowland of Nepal, it has expanded to higher altitudes in recent years. In 2025, Nepal recorded 5,196 confirmed dengue cases by late September across 75 districts, with Gandaki Province leading at 1,327 cases, followed by Bagmati (1,191) and Lumbini (911). Dengue has spread to higher altitudes and previously unaffected areas in Nepal, influenced by climate change and mosquito breeding patterns [5, 6]. Southeast Asia faces a substantial dengue burden, with over 140,000 cases and 437 deaths in 2025 alone, showing 40–75% increases over 2024 in countries like Thailand (53,961 + cases). Incidence rose significantly in the Philippines, Vietnam, Malaysia, and Singapore from 2000 to 2023, with post-COVID resurgence despite temporary pandemic declines. All four DENV serotypes circulate, exacerbated by warming temperatures and mobility.

Currently clinical dengue is classified as dengue with or without warning signs and severe dengue [7]. Warning signs as mentioned in WHO classification (2009) includes abdominal pain and tenderness, persistent vomiting, clinical fluid accumulation, mucosal bleed, lethargy, restlessness, liver enlargement > 2 cm and increase in hematocrit concurrent with rapid decrease in platelet count [7]. Common hemorrhagic manifestations in Dengue include petechiae, epistaxis, gum bleeding, gastrointestinal bleeding and hematuria [8].

## Objectives

In this study, we report laboratory and clinical profiles of adult dengue patients with epistaxis from a tertiary health care facility in Kathmandu, Nepal.

### Study design

In our study, 27 patients with dengue fever admitted to our tertiary center were evaluated between September 2022 and November 2024, all of whom were NS1-positive and presented with epistaxis. Admission was indicated due to the presence of warning signs and/or features of severe dengue, including active mucosal bleeding, significant thrombocytopenia, hemodynamic instability, deranged liver enzymes, and the need for close clinical and laboratory monitoring.

Gender predominance, prior comorbidities, previous medications, total hospital stay, other bleeding manifestations, platelet count and PT/INR on the day of epistaxis, AST/ALT levels and whether transfusion was required or not among these patients with epistaxis, were analyzed.

## Result

The average age of the patients was 42 ± 18.14 (range 14–90) years. Most of the patients (70.37%, 19/27) were male. Around half (48.15%) had some comorbidity. The most common comorbidity was Hypertension (HTN) which was seen in 25.93% (*n* = 7) patients and 11.11% (*n* = 3) patients had Diabetes Mellitus (DM). About 40% (*n* = 11) patients were on some drug prior to dengue infection for their comorbidity. Among the 11 patients (40.7%) receiving medications for pre-existing comorbidities, the most commonly used drugs were antihypertensive agents, oral hypoglycemic agents, insulin, and levothyroxine. None of the patients were receiving antiplatelet agents or anticoagulants prior to admission. The most common presenting complaint was fever seen in 85.19% patients (*n* = 23).

**Fig. 1:**
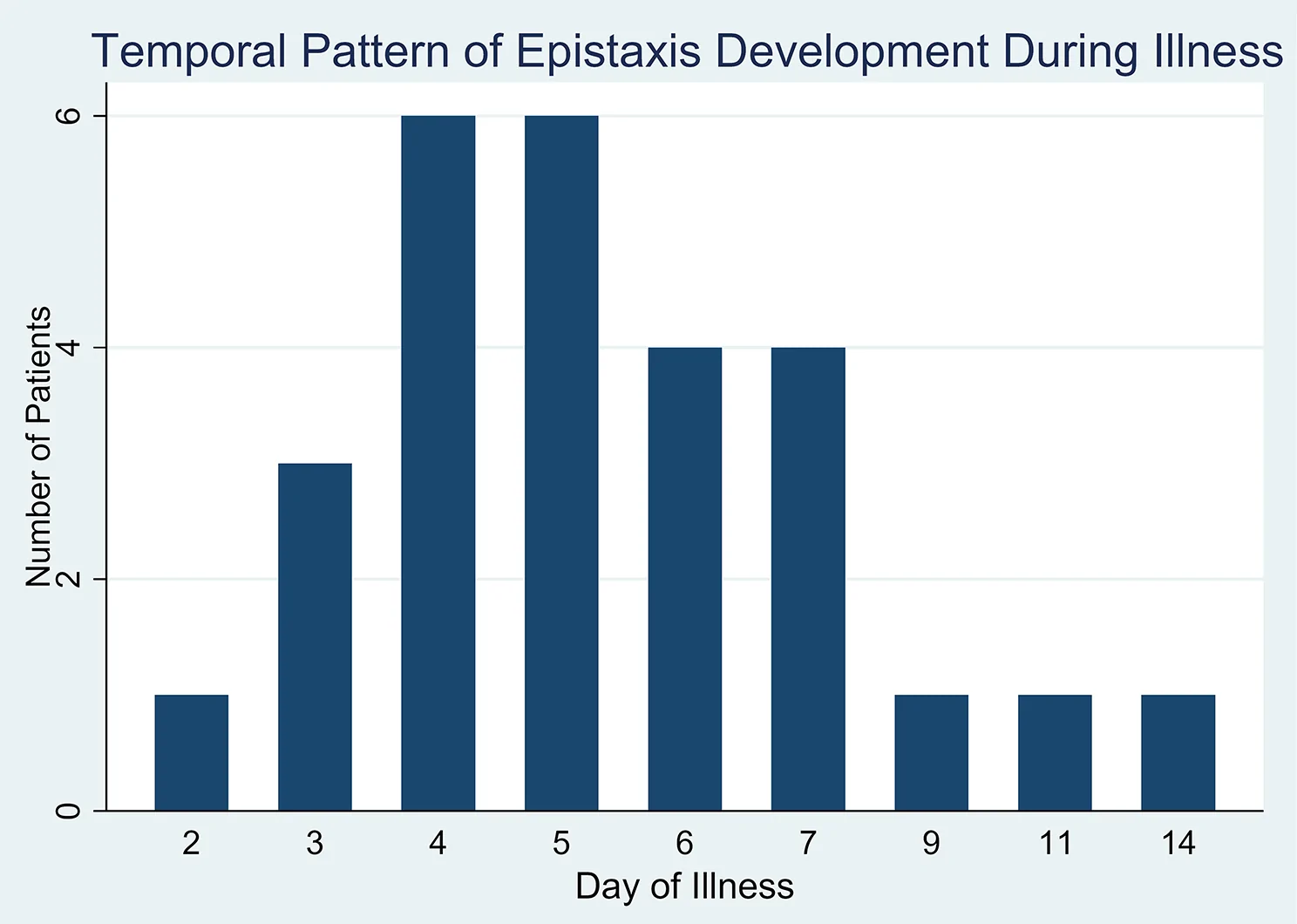
Temporal pattern of epistaxis onset during illness

**Fig. 2:**
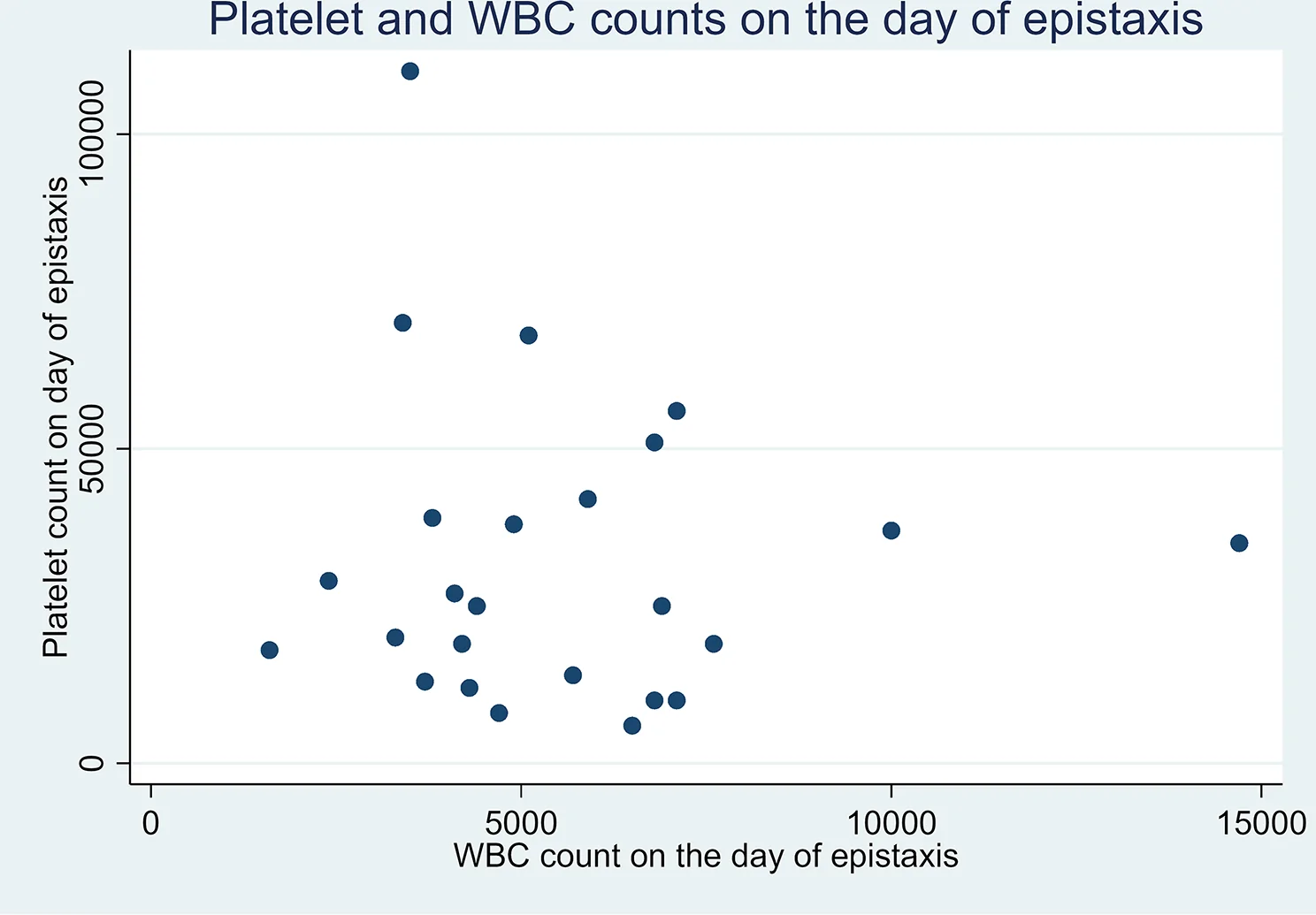
Platelet and WBC counts at the time of epistaxis

In this study, 59.26% patients had epistaxis at presentation while 40.74% developed epistaxis during hospital stay. The mean duration of illness at the time of epistaxis was 5.59 ± 2.56 days (range 2–14 days). The most common time of epistaxis was around 3–7 days (Fig. 2). Two thirds of the patients had no bleeding from other site except epistaxis. Of the one third who had bleeding from other sites, oral mucosal bleed was the most common present in 22.22% (*n* = 6) and Gastrointestinal (GI) bleeding was seen in 4 patients (14.82%). Among those who had bleeding from other sites, most were males (88.89%) (Fig. 1).

**Fig. 3:**
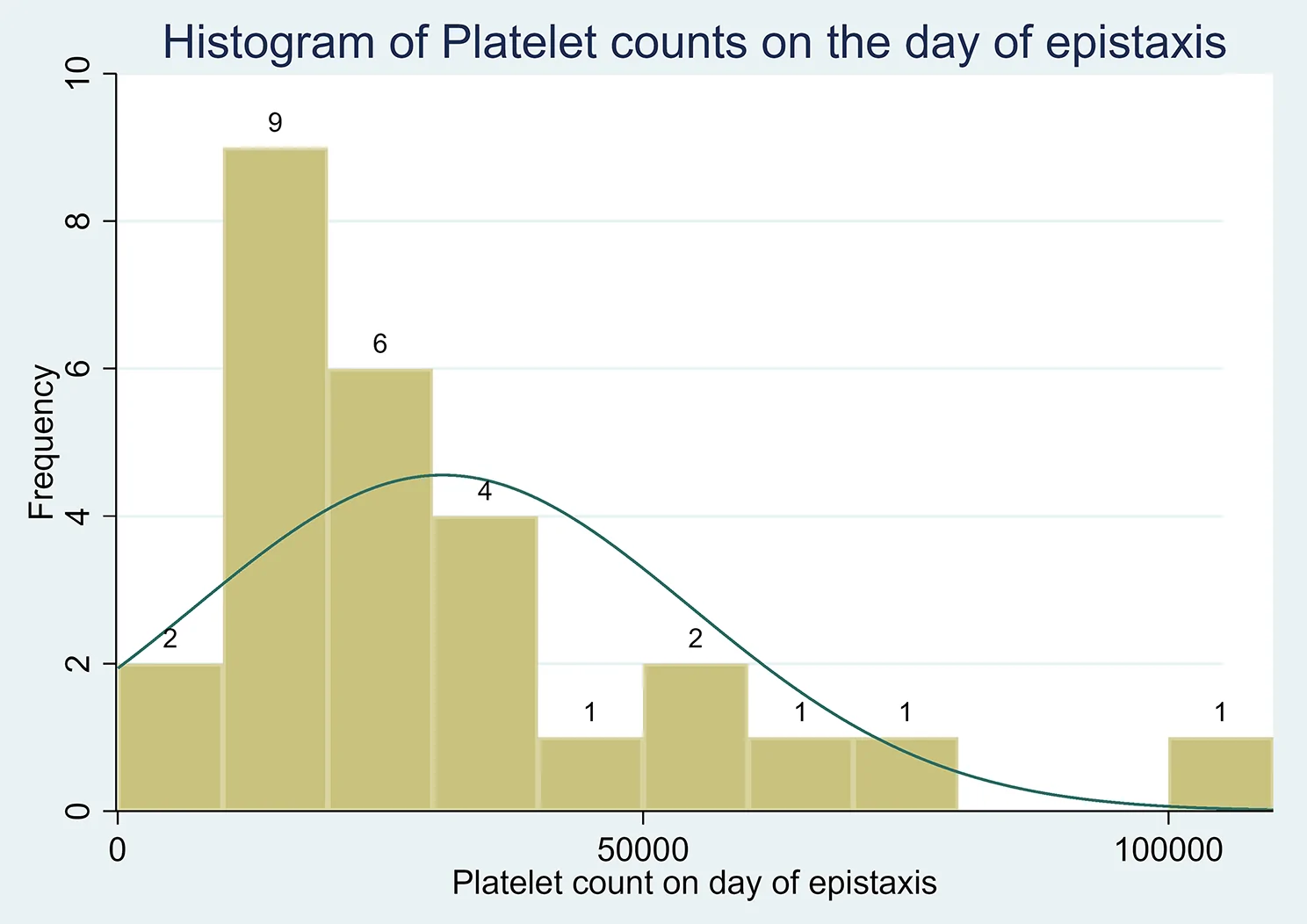
Histogram of platelet count on the day of epistaxis

The mean platelet count on the day of epistaxis was about 30,000 **/**µL (range 6000–110000 **/**µL). One patient had platelet more than 100,000**/**µL and on review the patient was a 14-year old child with lupus nephritis. She was not on antiplatelet agents or blood thinners. The mean hemoglobin (Hb) on the day of bleed was 13.77 ± 2.66 (range 9 -18.8). The mean WBC count on the day of bleed was around 5000 cells/µL (Figs. 2 and 3).

**Fig. 4:**
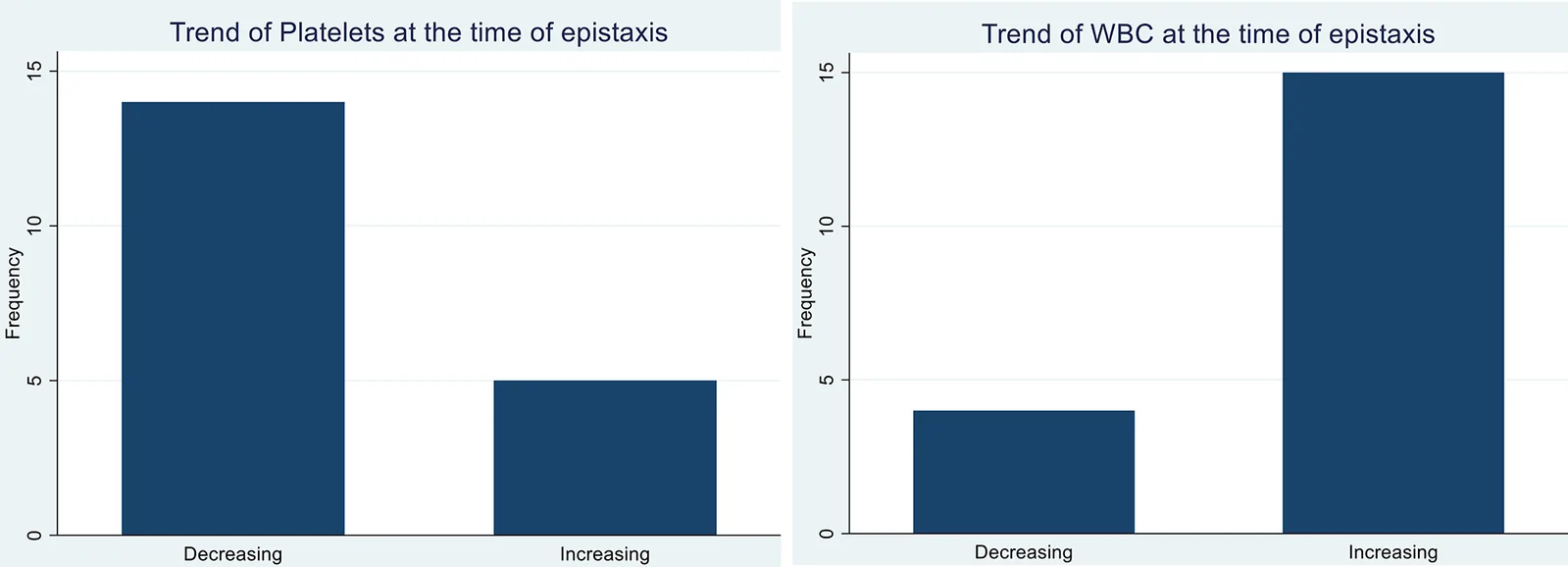
Trend of platelets and WBC at the time of epistaxis (*n* = 19 each)

At the time of epistaxis, most patients had increasing trend in WBC counts (55.56%). The trend could not be ascertained in 8 patients. Of those whose trend of WBC counts could be ascertained, 15/19 (78.95%) had increasing WBC counts. At the time of epistaxis, most patients had decreasing trend of platelets (51.85% of total, 73.68% of those whose trend could be ascertained) (Fig. 4).

AST and ALT levels on the day of epistaxis were elevated with a mean AST of 424 ± 615 and mean ALT of 166 ± 160 U/L. Among 27 patients, 62.96% (*n* = 17) required transfusion. 2 patients required Packed Red Blood Cells (PRBC) transfusion (1 patient required only PRBC while the other patient required Platelets and PRBC transfusion. The latter patient had esophageal ulcer and had presented with esophageal ulcer and melena. Among the 16 patients who needed platelet transfusion, the mean was 4.31 pints of Platelets Rich Plasma (PRP) (range 2–8 pints) (Table 1).

**Table 1:** Clinical and laboratory characteristics of dengue patients according to transfusion requirement

Factor	Level	No	Yes	*p*-value
Number		10	17	
Age, mean (SD)		34.70 (16.63)	46.29 (18.06)	0.11
Sex	F	5 (50%)	3 (18%)	0.075
	M	5 (50%)	14 (82%)	
Duration of hospital stay, mean (SD)		6 (4.47)	5.18 (2.83)	0.56
Day of illness at time of epistaxis, mean (SD)		5.10 (1.91)	5.88 (2.89)	0.45
Platelet count on day of epistaxis, mean (SD)		45,800 (30,268.429)	22,176.47(13,163.56)	0.009
Hemoglobin on day of bleed, mean (SD)		12.3 (2.49)	14.63 (2.43)	0.025
WBC count on day of bleed, mean (SD)		4870 (2352.90)	5811.76 (2761.99)	0.38

Among the 9 patients who had bleeding from other sites, 89% required transfusion and only 11% did not require transfusion. Platelet transfusions were administered in the form of random donor platelets (RDPs), as single donor platelets were not routinely available at our center during the study period. Among those who did not require transfusion were patients who had bleeding from gastrointestinal tract (melena, *n* = 1) and urinary tract (hematuria, *n* = 1). The majority of individuals who experienced bleeding from other sites were male, accounting for 88.89% (Table 2).

**Table 2:** Gender distribution among patients with and without bleeding from other sites. This table presents demographic, clinical, and hematological variables of patients stratified by whether transfusion was required or not

Other bleed	Sex		Total
	F	M	
None	7	11	18
Yes	1	8	9
Total	8	19	27

Mean duration of hospital stay was 5.48 ± 3.47 days (range 2–14 days). There was no mortality in these patients

## Discussion

This is a hospital-based case series study. 27 patients presenting with dengue fever disease from Sept 2022 to Nov 2024 were analyzed, in whom NS1 was positive and who simultaneously had epistaxis. These patients were admitted and treated at our tertiary center.

The age distribution of the study sample (*N* = 27) ranges from 14 to 90 years (mean = 42.0), indicating a wide age diversity among participants. These findings highlight that the sample is primarily composed of adults, though the presence of both younger (minimum = 14) and older individuals (maximum = 90) suggests diversity in age. Out of 27 patients, 70.4% (*n* = 19) were male and 29.6% (*n* = 8) were female.

In our study epistaxis occurred from the 2nd to the 14th day of illness with a mean of 5.6 days (SD = 2.56). Half of the patients experienced epistaxis by day 5, and 75% by day 7, suggesting a tendency for epistaxis to occur within the first week of illness. Dengue fever passes through 3 stages: the febrile stage lasting 2 to 7 days, the critical or leakage stage, which spans 24 to 48 h, and the recovery phase, which takes 2 to 7 days [9]. A study in India indicated that the hemorrhagic diathesis was a common complication of dengue fever and usually occurred toward the end of the febrile period, when the symptoms of classic dengue fever resolve. The study also found that bleeding was usually mild and manifested itself as epistaxis, gum bleeding, hematuria, and menorrhagia [10]. 

Out of 27 participants, 9 (33.3%) reported other types of bleeding in addition to epistaxis, which included mucosal bleed, gingival bleed, melena, hematemesis, hemoptysis and hematuria.

Prior studies done in India have reported bleeding in the form of purpura, petechiae, ecchymoses, gastrointestinal and retroperitoneal hemorrhage, rectus sheath hematoma, and mild to moderate bleeding from the skin, gums, nose, urinary tract, gastrointestinal tract, lungs, and uterus in dengue patients [10–14]. 

On the day of epistaxis, platelet count ranged from 6,000 to 110,000/µL, with a mean of about 30,000/µL, indicating substantial variability. The median platelet count was 25,000/µL, while two-thirds of the patients had platelets below 39,000/µL, indicating the predominance of moderate to severe thrombocytopenia. These findings highlight that most patients experienced epistaxis at relatively low platelet levels, supporting the role of thrombocytopenia in bleeding risk during illness. Despite a platelet count of 110,000, one of our patients had epistaxis, which may be attributed to underlying vascular fragility or immune-mediated coagulopathy associated with her history of systemic lupus nephritis.

Platelet count ranged from 12,000 to 150,000 with a mean of 30,760/µL in a clinical study conducted in India, which is quite similar to our findings [10]. The dengue virus has been isolated from various types of white blood cells, including polymorphonuclear leukocytes, monocyte/macrophages, and dendritic cells. It has also been detected in megakaryocyte progenitor cells and circulating platelets that are present in the bloodstream. This reflects that the dengue virus may cause a decrease in platelet count by directly interacting with megakaryocytes and platelets. Therefore, it is likely that two processes contribute to the development of thrombocytopenia in dengue patients: Impaired production of platelets and destruction of platelets in the peripheral regions [15, 16]. Possible mechanisms of low platelet counts in dengue are: disruption of platelet production in the bone marrow by the virus and acceleration of their destruction through activation and apoptosis. This delicate imbalance between reduced production and increased clearance reflects the body’s struggle against the infection [17]. 

Among the 27 patients, 51.85% (14 patients) had a decreasing platelet trend, suggesting a possible link between falling platelet levels and the occurrence of epistaxis. 18.52% (5 patients) showed an increasing trend, possibly indicating an early phase of recovery or a late-stage response. For 29.63% (8 patients), data on platelet trend was not available, limiting complete analysis. Our study found that most patients experienced a moderate level of thrombocytopenia, consistent with results from earlier research. Among the 17 patients involved in a study, all (100%) presented with epistaxis. Additionally, the study noted that a large number of patients showed signs of cutaneous bleeding, primarily in the form of rashes or petechiae [10]. 

Hemoglobin levels on the day of bleeding ranged from 9.0 to 18.8 g/dL, with a mean of 13.77 g/dL. A retrospective descriptive study conducted in Brazil showed anemia in 45.0% of the cases, with the lowest mean hemoglobin level of all patients who received any type of transfusion during hospitalization being 9.0 g/dL [18]. 

The WBC count on the day of epistaxis ranged from 1,600 to 14,700 cells/µL. The median was 4,900 cells/µL, indicating that half of the patients had either low normal or low WBC counts. Out of 27 patients, 55.56% (15 patients) showed an increasing WBC trend. The observed trend of rising WBC counts accompanied by a declining platelet count may point towards the nadir of platelet levels. This pattern aligns with the finding of Ananda Rao et al., who noted that platelet recovery typically follows the resurgence of WBCs [19]. This inverse trend could signify the critical turning point—where platelets are at their lowest and poised to rebound. In 29.63% (8 patients), the trend data was not available, which limits full interpretation.

On the day of epistaxis, the mean AST level was 424.4 IU/L (22–2170) IU/L and the mean ALT level was 166.7 IU/L (23–714) IU/L. Similar levels of ALT and AST were reported among dengue patients who presented with epistaxis or bleeding manifestations in Brazil, where more than 80% showed transaminase alterations, with AST ranging from 140.7 to 478.7 IU/L and ALT from 105.6 to 229.7 IU/L. Evidence suggests a link between elevated levels of these parameters and increased mortality. Association of these laboratory findings with liver dysfunction and acquired coagulopathy reflects the importance of closely monitoring coagulation status and considering the use of fresh frozen plasma (FFP) transfusions when necessary [18]. 

Among 17 patients who needed transfusion, the amount of PRP transfused ranged from 2 units (minimum) to 8 units (maximum) with an average of 4.31 units and a median of 4 units. Most patients (75%) received 5 units or less, and 25% received 3 units or less. On average 3–5 units of PRP were required per patient depending upon clinical severity and institutional protocols [20–22]. A study reported that 10.6% of patients with dengue hemorrhagic fever required some form of transfusion. Among these, 6.9% received platelet concentrates, 5% were given fresh frozen plasma (FFP), and 3.1% received packed red blood cells [9]. Nepal’s national dengue management guidelines emphasize judicious fluid therapy as the foundation of dengue care and recommend reserving platelet transfusion for patients with significant hemorrhagic manifestations rather than for isolated thrombocytopenia.

This study has several limitations. It was a retrospective, single-center study with a small sample size, limiting the generalizability of the findings. The inclusion of only hospitalized dengue patients with epistaxis may have introduced selection bias, and incomplete serial laboratory data limited trend analysis in some patients. Additionally, the absence of a comparison group precludes assessment of a causal relationship between epistaxis and disease severity. Larger prospective multicenter studies are needed to validate these findings and clarify the prognostic significance of epistaxis in dengue.

## Conclusion

In this hospital-based case series, epistaxis most frequently occurred during the critical phase of dengue (days 3–7 of illness) and was associated with severe thrombocytopenia, dynamic changes in leukocyte counts, and elevated liver transaminases. Patients with additional bleeding manifestations required transfusion more frequently, reflecting a greater burden of hemorrhagic complications. These findings highlight the need for careful clinical evaluation and serial laboratory monitoring of dengue patients presenting with epistaxis, particularly during the critical phase, to facilitate timely recognition and management of bleeding complications. Given the retrospective design, small sample size, and lack of a comparison group, the findings should be interpreted as descriptive. Prospective multicentre studies are needed to determine the prognostic significance of epistaxis and to refine evidence-based transfusion strategies in dengue.

## Data Availability

The datasets used during the study are available from corresponding author on reasonable request.

## Abbreviations

WBC: Whole Blood Count
AST: Aspartate Transaminase
ALT: Alanine Transaminase
WHO: World Health Organization
PT: Prothrombin Time
INR: International Normalized ratio
PRBC: Packed Red Blood Cells
DM: Diabetes Mellitus
GI: Gastro-intestinal

## References

[1] Pierson TC, Kielian M. Flaviviruses: braking the entering. Curr Opin Virol. 2013;3(1):3–12. 10.1016/j.coviro.2012.12.001 PMC3601797 23352692

[2] Wong JM, Adams LE, Durbin AP, Muñoz-Jordán JL, Poehling KA, Sánchez-González LM, et al. Dengue: a growing problem with new interventions. Pediatrics [Internet]. 2022 May 11 [cited 2022 Jun 27];149(6):e2021055522. Available from: https://publications.aap.org/pediatrics/article/149/6/e2021055522/187012/Dengue-A-Growing-Problem-With-New-Interventions. 10.1542/peds.2021-055522 35543085

[3] Witte P, Venturini S, Meyer H, Zeller A, Christ M. Dengue fever—diagnosis, risk stratification, and treatment. PubMed. 2024;(Forthcoming). 10.3238/arztebl.m2024.0175 PMC12036110 39297280

[4] Bhandari S, Blackburn JK, Ryan SJ. Spatial patterns of dengue incidence in Nepal during record outbreaks in 2022 and 2023: implications for public health interventions. medRxiv (Cold Spring Harbor Laboratory; 2024. 10.4269/ajtmh.24-0747 PMC12360065 40494337

[5] Tuladhar R, Singh A, Varma A, Choudhary DK. Climatic factors influencing dengue incidence in an epidemic area of Nepal. BMC Res Notes. 2019 Mar 13 [cited 2019 Jun 16];12(1). Available from: https://bmcresnotes.biomedcentral.com/articles/10.1186/s13104-019-4185-4. 10.1186/s13104-019-4185-4 PMC6417253 30867027

[6] Dhimal M, Gautam I, Kreß A, Müller R, Kuch U. PJ <>Lammie editor 2014 Spatio-Temporal Distribution of Dengue and Lymphatic Filariasis Vectors along an Altitudinal Transect in Central Nepal. PLoS Negl Trop Dis 8 7 e3035. 10.1371/journal.pntd.0003035 PMC4117448 25078276

[7] Srikiatkhachorn A, Rothman AL, Gibbons RV, Sittisombut N, Malasit P, Ennis FA, et al. Reply to Akbar et al. Clin Infect Dis. 2012;54(12):1821–2.

[8] Rao SV, Jacob GG, Raju NA, Ancheri SA. Spontaneous arterial hemorrhage as a complication of dengue. Indian J Crit Care Med. 2016;20(5):302–4. 10.4103/0972-5229.182201 PMC4876654 27275081

[9] Kalayanarooj S. Clinical manifestations and management of dengue/DHF/DSS. Trop Med Health [Internet]. 2011 [cited 2019 Mar 27];39(4 Suppl):S83–7. Available from: https://www.ncbi.nlm.nih.gov/pmc/articles/PMC3317599/. 10.2149/tmh.2011-S10 PMC3317599 22500140

[10] Tabassum Khan N. Dengue haemorrhagic fever (Dhf). Arch Med Case Rep Case Study. 2022;6(1):1–3.

[11] Thapa B, Pandey A, Santosh Gautum, Kc S, Prabha D, Chhetri E, Pokhrel, et al. Clinicopathological Profile of Dengue Infection in a Tertiary Care Centre in Nepal. J Nepal Health Res Counc. 2023;20(4):859–67. 10.33314/jnhrc.v20i4.4172 37489668

[12] Lee IK, Liu JW, Yang KD. Fatal dengue hemorrhagic fever in adults: emphasizing the evolutionary pre-fatal clinical and laboratory manifestations. Halstead SB, editor. PLoS Neglected Trop Dis. 2012;6(2):e1532. 10.1371/journal.pntd.0001532 PMC3283557 22363829

[13] Chandima Jeewandara, Gomes L, Wickramasinghe NC, Gutowska-Owsiak D, Waithe D, Paranavitane SA, et al. Platelet activating factor contributes to vascular leak in acute dengue infection. 2015 Feb 3 [cited 2023 Jun 4];9(2):e0003459–9. Available from: https://www.ncbi.nlm.nih.gov/pmc/articles/PMC4315531/. 10.1371/journal.pntd.0003459 PMC4315531 25646838

[14] Orsi FA, Angerami RN, Mazetto BM, Quaino SK, Santiago-Bassora F, Castro V, et al. Reduced thrombin formation and excessive fibrinolysis are associated with bleeding complications in patients with dengue fever: a case–control study comparing dengue fever patients with and without bleeding manifestations. BMC Infect Dis. 2013;13(1). 10.1186/1471-2334-13-350 PMC3733705 23890510

[15] Kusama Y, Ito K, Tajima S, Kutsuna S. A pediatric case of imported dengue hemorrhagic fever in Japan. J Gen Family Med. 2017;18(6):414–7. 10.1002/jgf2.83 PMC5729368 29264075

[16] Sreenivasulu T, Jahnavi K. A study of clinical profile of patients with Dengue fever at a tertiary care hospital. Int J Adv Med. 2018;5(1):202–2.

[17] Khazali AS, Hadrawi WH, Ibrahim F, Othman S, Nor Rashid N. Thrombocytopenia in dengue infection: mechanisms and a potential application. Expert Rev Mol Med [Internet]. 2024 Oct 14 [cited 2026 Jul 29];26:e26. https://pubmed.ncbi.nlm.nih.gov/39397710/. 10.1017/erm.2024.18 PMC11488332 39397710

[18] Fujimoto DE, Koifman S. Clinical and laboratory characteristics of patients with dengue hemorrhagic fever manifestations and their transfusion profile. Revista Brasileira de Hematologia e Hemoterapia. 2014;36(2):115–20. 10.5581/1516-8484.20140027 PMC4005509 24790536

[19] Ananda Rao A, U RR, Gosavi S, Menon S. Dengue fever: prognostic insights from a complete blood count. Cureus. 2020. 10.7759/cureus.11594 PMC7752744 33364116

[20] Tayal A, Kabra SK, Lodha R. Management of dengue: an updated review. Indian J Pediatr. 2022;90(2). 10.1007/s12098-022-04394-8 PMC9793358 36574088

[21] Jorge CF, Nakazaki, Cotera-Ramón AI. Assessment of the importance of platelet transfusion in patients with severe dengue: a systematic review. Iberoamerican J Med. 2024;6(2):69–77.

[22] Jun D, Setiawan VW, Ng CH, Lim WH, Muthiah M, Tan S, et al. Global burden of liver cancer in males and females: Changing etiological basis and the growing contribution of NASH. Hepatology. 2022;77(4):1150–63. 10.1002/hep.32758 36037274

